# Expression of STAT3-regulated genes in circulating CD4+ T cells discriminates rheumatoid arthritis independently of clinical parameters in early arthritis

**DOI:** 10.1093/rheumatology/kez003

**Published:** 2019-02-08

**Authors:** Amy E Anderson, Nicola J Maney, Nisha Nair, Dennis W Lendrem, Andrew J Skelton, Julie Diboll, Philip M Brown, Graham R Smith, Ruaidhrí J Carmody, Anne Barton, John D Isaacs, Arthur G Pratt

**Affiliations:** 1 Musculoskeletal Research Group, Institute of Cellular Medicine, Newcastle University, Newcastle upon Tyne, UK; 2 Arthritis Research UK Centre for Genetics and Genomics, Centre for Musculoskeletal Research, and NIHR Manchester Musculoskeletal Biomedical Research Centre, Manchester NHS Foundation Trust, Manchester, UK; 3 Bioinformatics Support Unit, Faculty of Medical Sciences, Newcastle University, UK; 4 Newcastle upon Tyne Hospitals NHS Foundation Trust, Newcastle upon Tyne, UK; 5 Centre for Immunobiology, Institute of Infection, Immunity, and Inflammation, University of Glasgow, Glasgow, UK

**Keywords:** rheumatoid arthritis, T lymphocytes, gene expression

## Abstract

**Objectives:**

Dysregulated signal transduction and activator of transcription-3 (STAT3) signalling in CD4+ T cells has been proposed as an early pathophysiological event in RA. We sought further evidence for this observation, and to determine its clinical relevance.

**Methods:**

Microarray technology was used to measure gene expression in purified peripheral blood CD4+ T cells from treatment-naïve RA patients and disease controls newly recruited from an early arthritis clinic. Analysis focused on 12 previously proposed transcripts, and concurrent STAT3 pathway activation was determined in the same cells by flow cytometry. A pooled analysis of previous and current gene expression findings incorporated detailed clinical parameters and employed multivariate analysis.

**Results:**

In an independent cohort of 161 patients, expression of 11 of 12 proposed signature genes differed significantly between RA patients and controls, robustly validating the earlier findings. Differential regulation was most pronounced for the STAT3 target genes *PIM1*, *BCL3* and *SOCS3* (>1.3-fold difference; *P* < 0.005), each of whose expression correlated strongly with paired intracellular phospho-STAT3. In a meta-analysis of 279 patients the same three genes accounted for the majority of the signature’s ability to discriminate RA patients, which was found to be independent of age, joint involvement or acute phase response.

**Conclusion:**

The STAT3-mediated dysregulation of *BCL3*, *SOCS3* and *PIM1* in circulating CD4+ T cells is a discriminatory feature of early RA that occurs independently of acute phase response. The mechanistic and functional implications of this observation at a cellular level warrant clarification.

**Rheumatology key messages**

- Three STAT3-regulated genes discriminate RA independently of clinical parameters in early arthritis CD4+ lymphocytes.
- The mechanistic relevance of these genes’ activation amongst early RA CD4+ lymphocytes awaits clarification.

## Introduction

RA is a chronic disease of immune dysregulation the pathogenesis of which remains incompletely understood [1]. An orchestrating role for CD4+ T cells is suggested by a number of lines of evidence, including accumulating data from genetic association studies [2, 3], analyses of diseased synovia [4] and the observed therapeutic efficacy of co-stimulation blockade [5]. We previously identified a 12-gene CD4+ T cell expression signature in early arthritis patients that predicted a diagnosis of RA [6]. This signature comprised an over-representation of genes regulated by signal transduction and activator of transcription-3 (STAT3), each of whose expression correlated with paired serum levels of IL-6, itself a prominent inducer of STAT3 signalling [7]. Using flow cytometry we recently confirmed the importance of IL-6-mediated STAT3 activation in CD4+ T cells (in contrast to other circulating cytokines and leukocytes) as an early event in the clinical phase of RA, and suggested its potential value as a diagnostic biomarker [8].

Aberrant STAT3 signalling has a well-documented role in tumorigenesis via induction of pro-survival and cell cycle pathways [9–11]. Our observations add to accumulating evidence that analogous mechanisms of STAT3 dysregulation might sustain autoimmunity [12, 13]. Through a more sophisticated understanding of IL-6/STAT3 signalling at a cellular level, therapies that go beyond generic blockade of the IL-6 inflammatory cascade, instead targeting disease-specific mechanisms, may be uncovered [14].

The current investigation sought to validate the relevance of our previously described CD4+ T cell gene signature in a distinct early arthritis cohort. In particular, we determined the extent to which expression of STAT3-regulated genes was independently associated with a diagnosis of RA when considered alongside clinical parameters such as inflammation.

## Methods

### Patients

During 2012–13, consecutive patients were recruited from the Newcastle Early Arthritis Cohort, which has been described in detail elsewhere [6, 15, 16], and peripheral blood was obtained prior to commencement of therapy. Initial diagnoses were validated at follow-up visits over a median period of 20 months (range 13–25) as described [8], and with reference to 2010 ACR/EULAR classification criteria for RA [17]. All patients gave written, informed consent for inclusion into the study, which was approved by the local Regional Ethics Committee.

### Twelve-gene expression signature measurement in CD4+ T cells of the independent cohort

Total RNA was extracted from CD4+ T cells positively selected from monocyte-depleted whole blood within 4 h of blood draw as previously described [6]. cRNA generated from 250 ng total RNA (Illumina TotalPrep RNA Amplification Kit) was hybridized to the Illumina Human HT12v4 BeadChip (Illumina, San Diego, CA, USA). After quality control using established methods previously outlined [6], data relating exclusively to the 12 signature genes previously identified [6] were extracted for detailed analysis. Expression data used for this experiment are available in the Gene Expression Omnibus database (GEO: http://www.ncbi.nlm.nih.gov/geo; accession number GSE80513). Since the HT12v4 BeadChip annotation differed slightly from the WG6v3 array used for our original work, unique Illumina *NuId* references were used instead to map probes of identical sequence for this purpose.

### Flow cytometric determination of STAT3 pathway activation in CD4+ T cells

Phosflow cytometry was performed on freshly drawn, unstimulated whole blood obtained contemporaneously with that used for CD4+ T cell RNA extraction. Anti-CD4-APC-eFluor 780 (SK3) (eBioscience Ltd, Hatfield, UK), anti-Stat3 (pY705)-Alexa Fluor 647 (4/P-STAT3) and anti-CD3-Pacific Blue (UCHT1) (both BD Biosciences, Oxford, UK) were used along with appropriate buffers and controls in the staining protocol as previously described [8]. Data were collected on a BD FACSCanto II (BD Biosciences, Oxford, UK) and analysed using FlowJo (Treestar, Ashland, OR, USA).

### Combined cohort microarray analysis

Since baseline and follow-up diagnostic classification of patients in the previously described cohort [6] was undertaken with reference to the 1987 ACR criteria [18], retrospective application of the 2010 ACR/EULAR classification criteria was applied [17], so that both cohorts were similarly classified. Thirteen of 62 patients previously classified at baseline with undifferentiated arthritis became 2010-RA, and 6 of 47 1987-RA patients did not fulfil the 2010 criteria. Next, a *de novo* pipeline for the normalization and quality control of independently derived raw microarray datasets from the previous and current patient cohorts (GEO accession numbers GSE20098 and GSE80513, respectively) was employed as previously described, demonstrably accounting for anticipated batch effects [19].

### Statistical analysis

Hierarchical clustering (Euclidian distance metric; Ward’s linkage method) was performed and visualized in R programming environment (https://r-project.org). Mann–Whitney U and Kruskal Wallis tests were used for two-group and multiple group univariate analyses, respectively, along with chi-squared (χ^2^) and Komogorov-Smirnov tests as indicated in the text. Bivariate correlations were determined using Spearman’s Rho, and logistic regression was used for multivariate analyses with validated diagnostic outcome as the dependent variable, and independent variables as detailed in the text. Receiver-operating characteristic curves for competing logistic regression models were constructed and differences in their areas under the curve compared using t-tests. In addition, scatterplots overlaid with non-parametric density plots [20] were used to depict separation of comparator groups attributable to normalized gene expression, using SAS Institute JMP statistical visualization software (version 13; Cary, NC, USA).

## Results

### Baseline clinical characteristics of newly recruited patients

Some 161 early arthritis patients were enrolled into the study, of whom 47 (29%) were diagnosed with RA and the remainder with alternative diagnoses; their baseline clinical characteristics are summarized in Table 1. Early RA patients differed, on average, from other early arthritis clinic attendees by a higher acute phase response, more swollen and tender joints, circulating autoantibodies (RF ACPA) and older age.

**Table 1:** Baseline clinical characteristics of patients according to diagnosis

	Diagnosis^a^		*P*-value^b^
	RA (*n* = 47)	Non-RA (*n* = 114)	
Age, years	60 (21–87)	51 (17–92)	<0.001
% Female	74	68	ns
Symptom duration	12 (2–52)	12 (2–>52)	ns
TJC28	6 (1–20)	2 (0–25)	0.001
SJC28	1 (1–17)	0 (0–9)	0.002
CRP, g/l	10 (<5–66)	5 (<5–189)	0.005
ESR	21 (4–86)	9 (1–113)	0.004
%RF+	64	10	<0.001
%ACPA+	64	0	<0.001
DAS28	4.32 (2.24–7.15)	n/a	n/a
Non-RA diagnoses, number (% of 114)		PsA, 20 (17) Other SpA, 18 (16) Crystal, 10 (9) Other IA, 8 (7) OA, 25 (22) Other non- IA, 33 (29)	

Values are median (range) unless otherwise stated.

a Baseline diagnosis was confirmed at median 20 months (range 13–25).

b Mann–Whitney U test or chi-squared test with Yates’ continuity correction for continuous and dichotomous data respectively. TJC: tender joint count; SJC: swollen joint count; ns: not significant; n/a: not applicable; IA: inflammatory arthritis.

### Independent validation of STAT3-regulated CD4+ T cell signature in early RA

**Fig. 1:**
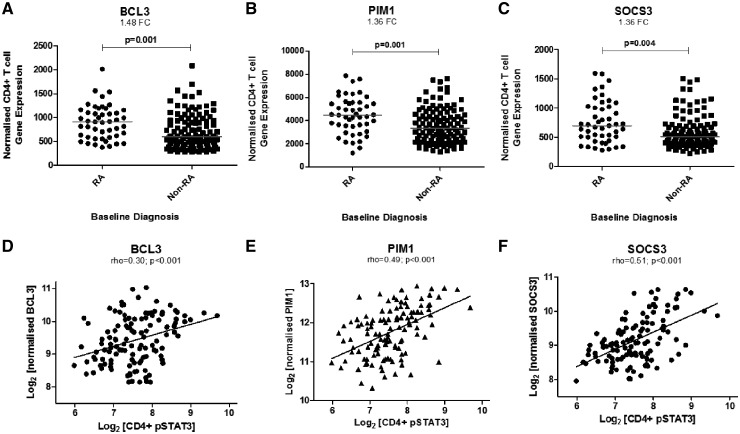
Expression of STAT3-regulated genes in circulating CD4+ lymphocytes of an independent early arthritis cohort (**A**–**C**) Normalized gene expression of three STAT3-regulated genes in circulating CD4+ T cells of an independent early arthritis cohort of 161 patients presenting with RA or other arthritides (Non-RA). Mann–Whitney U tests used to determine *P*-values; FC denotes fold-change. (**D**–**F**) Bivariate correlation between the same genes’ normalized expression and pSTAT3 measurements in paired circulating CD4+ T cells of early arthritis clinic attendees. Spearman’s Rho correlation coefficients and associated *P*-values are depicted. STAT3: signal transduction and activator of transcription-3; pSTAT: phospho-STAT3.

In our independent cohort of 161 treatment-naïve early arthritis clinic attendees, significant differences in normalized expression were seen for 11 of the 12 previously identified signature genes between RA and non-RA CD4+ T cells (Table 2). Only thee (*PIM1*, *BCL3* and *SOCS3*) achieved the 1.2-fold difference between comparator groups set as a threshold in our original study [6], but they did so comfortably with >1.3-fold differences being observed in each case (Table 2 and Fig. 1A–C). Indeed, it was notable that these three genes were ranked amongst the top 25 differentially expressed by fold-difference out of a total of 30 458 non-redundant, filtered probes in the source microarray dataset, something that would be highly unlikely to have occurred by chance (*P* = 9.8 × 10^−10^, one-sample Kolmogorov-Smirnov test). The three highlighted genes are known to be regulated by STAT3 [21–23]. We therefore hypothesized that their normalized expression would in turn depend upon constitutive STAT3 phosphorylation in CD4+ T cells, as measured using flow cytometry of contemporaneously obtained fresh blood samples. Intracellular phospho-STAT3 measurements indeed correlated strikingly with paired *BCL3*, *SOCS3* and *PIM1* gene expression in *ex vivo* CD4+ T cells of early arthritis patients (Fig. 1D–F), but not with that of other genes in the signature such as *PDCD1* or *IGFL2*, which are not known to be induced by STAT3 (supplementary Fig. S1, available at *Rheumatology* online). These data confirm the importance of STAT3 signalling as a mediator of *BCL3*, S*OCS3* and *PIM1* gene induction in early RA.

**Table 2:** Normalized expression values of indicated transcripts in early arthritis patient diagnostic groups of the independent cohort

Gene			Normalized expression^a^			
Symbol	RefSeq	Illumina Probe ID	RA (*n* = 47)	Non-RA (*n* = 114)	Fold-change^b^	*P*-value^c^
*BCL3*	**NM_005178**	**ILMN_1710514**	**867**	**585**	**1.48**	**<0.001**
*PIM1*	**NM_002648**	**ILMN_1815023**	**3567**	**2633**	**1.35**	**<0.001**
*SOCS3*	**NM_003955**	**ILMN_1781001**	**850**	**614**	**1.38**	**0.004**
*LDHA*	NM_005566	ILMN_1807106	7493	6572	1.14	<0.001
*GPRIN3*	CR743148^d^	ILMN_1901616	377	322	1.17	0.004
*MUC1*	NM_001044391	ILMN_1756992	310	283	1.10	0.013
*PDCD1*	NM_005018	ILMN_1806725	168	159	1.06	0.029
*SBNO2*	NM_014963	ILMN_1808811	193	180	1.07	0.011
*IGFL2*	NM_001002915	ILMN_1790227	142	135	1.05	0.017
*LOC731186*	XM_001128760	ILMN_1900154	148	145	1.02	0.033
*CMAHP*	NR_002174	ILMN_1704084	222	217	1.02	ns
*NOG*	NM_005450	ILMN_1652287	143	150	−1.05	0.007

RefSeq accession numbers and Illumina probe IDs are given (see supplementary Table S1, available at *Rheumatology* online for probe sequences).

a Median normalized gene expression values presented.

b Linearized fold-change relative to non-RA group given.

c Mann–Whitney U test.

d Transcript CR743148 has been retired from the National Center for Biotechnology Information, but the expressed sequence tag corresponds to splice variant(s) within the *GPRIN3* gene (chromosome 4.90). Genes demonstrating >1.2-fold differences indicated in boldface. ns: not significant.

### Twelve-gene signature’s ability to discriminate early RA in combined cohort accounted for by a three-gene subset

**Fig. 2:**
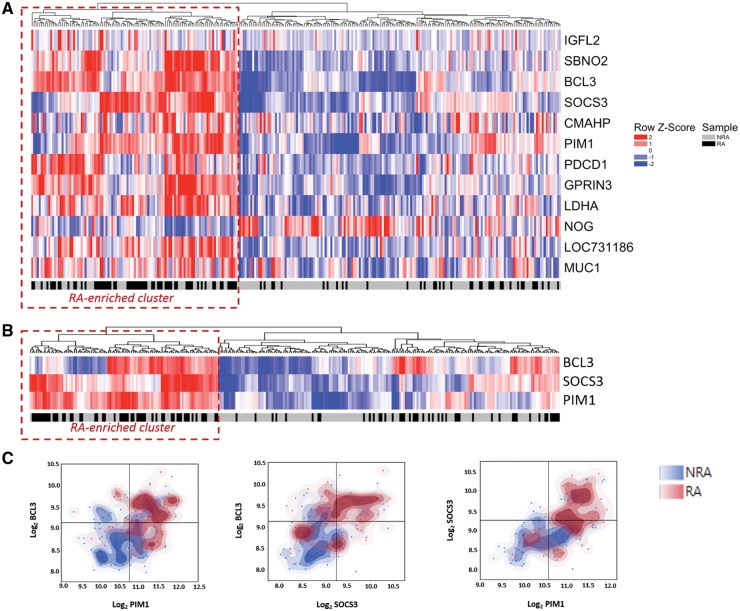
Discriminatory utility of a proposed CD4+ lymphocyte signature is primarily accounted for by three genes (**A**) Dendrogram and heat map depicting results of hierarchical clustering of 279 early arthritis patients (columns) according to normalized expression of 12 signature genes in circulating CD4+ T cells (rows). Red dotted line identifies RA-enriched cluster highlighted in text. (**B**) Analogous result as for (A), based on normalized expression levels of three-gene signature only. (**C**) Scatterplots overlaid with non-parametric density plots separate RA patients and non-RA patients based on normalized expression alone, such that the two populations in each case preferentially occupy the top right and bottom left quadrants, respectively.

Our previously described 12-gene CD4+ T cell signature was originally identified in untreated early RA patients defined prior to the publication of modified classification criteria for the condition. In our independent RA cohort, defined under the new classification system, a subset of three genes (*BCL3*, *SOCS3* and *PIM1*) was clearly up-regulated. To investigate whether this held true following diagnostic re-classification of the previous cohort, and to increase the statistical power of our study, a pooled analysis of microarray data was carried out. Some 101 of 279 (36%) in the combined cohort were diagnosed with RA, and these individuals were again distinguishable from other early arthritis clinic attendees at baseline by their older age, increased number of tender and swollen joints, and higher acute phase responses; detailed characteristics of the combined cohort are summarized in supplementary Table S1, available at *Rheumatology* online*.*Fig. 2A depicts the results of hierarchical clustering based on the 12-gene signature in the combined cohort, discriminating a subset of individuals enriched for a diagnosis of RA [includes 68/101 (67%) RA patients in the overall population compared with 40/178 (22%) non-RA patients; *P* < 0.001, χ^2^ test]. Again, only the expression of *BCL3*, *SOCS3* and *PIM1* stood out as being >1.2-fold up-regulated in early RA on univariate analysis of this combined cohort (supplementary Table S2, available at *Rheumatology* online). Hierarchical clustering confirmed that these three genes alone accounted for the majority of the previously noted clustering effect, once more discriminating a subgroup that was significantly enriched for RA (χ^2^*P* < 0.001; Fig. 2B). Moreover, segregation of RA patients from disease controls is evident when representing the data in 2D space according to their normalized expression (Fig. 2C). These data indicate that *BCL3*, *SOCS3* and *PIM1* account for the majority of the previously described 12-gene signature’s discriminatory ability with respect to a diagnosis of RA in the setting of an early arthritis clinic.

### Association of gene expression with RA is independent of baseline clinical parameters

**Fig. 3:**
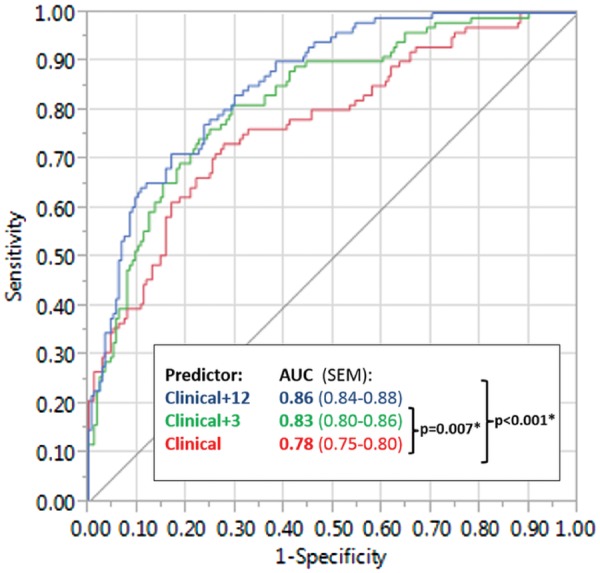
CD4+ lymphocyte gene signatures add independent diagnostic value to clinical parameters Receiver-operating characteristic curves depicting the extent to which consideration of the 12-gene and three-gene signatures (blue and green lines, respectively) add independent discriminatory value to five clinical parameters (age, swollen and tender joint count, CRP and ESR; red line), with respect to a diagnosis of RA *vs* non-RA (see text). AUC: area under curve.

Considering the potentially confounding influence of age, swollen or tender joint count, and, in particular, acute phase response (Table 1 and supplementary Tables S1 and S3, available at *Rheumatology* online), we used logistic regression to confirm that the up-regulation of *BCL3*, *PIM1* and *SOCS3* observed in early RA was independent of these clinical parameters (*P* < 0.05 for each gene; Table 3). To quantify the relative additive value of a three-gene (comprising *BCL3*, *SOCS3* and *PIM1*) or 12-gene signature over clinical parameters alone, results of multivariate analyses summarized as composite receiver-operating characteristic curves were compared (Fig. 3). Hence, by including the 12-gene signature in a model that included age, swollen joint count, tender joint count, CRP and ESR, a statistically significant area under the curve increase from 0.78–0.87 was achieved (*P* < 0.001). Interestingly, however, the three-gene signature accounted for a substantial component of this effect (area under the curve increase 0.78–0.83; *P* = 0.007). Considered together, these findings suggest that up-regulated expression of *BCL3*, *SOCS3* and *PIM1* in circulating CD4+ T cells of early RA patients is independent of potentially confounding clinical parameters, and accounts for much of the previously described 12-gene signature’s discriminatory ability for early RA.

**Table 3:** Summaries of logistic regression outputs in respect of RA vs non-RA diagnoses

	B	SE	Wald	df	*P*-value	Exp[B] (95% CI)
**BCL3**	**0.001**	**0.001**	**6.964**	**1**	**0.008**	**1.001** (1.000–1.002)
Age	0.037	0.011	11.831	1	0.001	1.034 (1.016–1.059)
CRP	−0.005	0.006	0.546	1	0.460	0.995 (0.983–1.008)
ESR	0.007	0.007	1.001	1	0.317	1.007 (0.993–1.022)
TJC28	0.034	0.020	2.967	1	0.085	1.034 (0.995–1.075)
SJC28	0.227	0.059	14.860	1	<0.001	1.255 (1.118–1.409)
Constant	−4.399	0.728	36.494	1	<0.001	0.012

**PIM1**	**0.000**	**0.000**	**7.996**	**1**	**0.005**	**1.000** (1.000–1.001)
Age	0.034	0.011	10.258	1	0.001	1.034 (1.013–1.056)
CRP	−0.006	0.006	1.019	1	0.313	0.994 (0.981–1.006)
ESR	0.008	0.007	1.203	1	0.273	1.008 (0.994–1.022)
TJC28	0.036	0.020	3.320	1	0.068	1.036 (0.997–1.077)
SJC28	0.214	0.058	13.621	1	<0.001	1.239 (1.106–1.388)
Constant	−4.512	0.734	37.843	1	<0.001	0.011

**SOCS3**	**0.001**	**0.001**	**5.930**	**1**	**0.015**	**1.001** (1.000–1.002)
Age	0.038	0.011	12.479	1	<0.001	1.038 (1.017–1.060)
CRP	−0.007	0.006	1.186	1	0.276	0.993 (0.981–1.006)
ESR	0.009	0.007	1.648	1	0.199	1.009 (0.995–1.024)
TJC28	0.031	0.019	2.587	1	0.108	1.032 (0.993–1.072)
SJC28	0.150	0.082	3.327	1	0.068	1.162 (0.989–1.365)
Constant	−4.424	0.737	36.009	1	<0.001	0.012

*BCL3*, *PIM1* and *SOCS3* (boldface) are, respectively, considered as independent candidate variables alongside age, CRP, ESR, TJC and SJC. TJC: tender joint count; SJC: swollen joint count.

## Discussion

In this validation study, we confirmed the ability of our previously proposed CD4+ T cell gene expression signature to identify RA patients amongst unselected, treatment-naïve early arthritis clinic attendees. Normalized gene expression differed significantly between RA patients and disease controls for 11 out of the 12 signature genes, but the fold-changes were most striking for *BCL3*, *PIM1* and *SOCS3*. Each of these genes is known to be regulated by STAT3, and their expression correlated significantly with paired CD4+ T cell phospho-STAT3 levels. Indeed, hierarchical clustering suggested that the ability of the 12-gene signature to discriminate RA patients in this replication cohort was almost entirely accounted for by these three genes alone. The reproducibility of this component of the original gene signature in two independent studies separated by 5 years is remarkable given the heterogeneity of the patient population presenting to early arthritis clinics. Furthermore, the signature is robust to replacement of 1987 ACR classification criteria [18] used to define RA in our previous analysis with updated criteria developed for use specifically in the setting of early disease [17]. Finally, our analysis demonstrated that the associations of *BCL3*, *PIM1* and *SOCS3* gene expression with diagnostic outcome are independent of clinical parameters such as age and systemic inflammation. Rather than being mere bystander phenomena, our data could indicate a direct role for STAT3-regulated gene induction in RA pathogenesis, for example via altered T cell effector function. This possibility remains the subject of ongoing investigation.

A growing body of evidence now highlights IL-6-mediated dysregulation of CD4+ T cell STAT3 signalling during RA development [24, 25]. Amongst RA patients who experience good therapeutic responses to the anti-IL-6 receptor monoclonal antibody tocilizumab, concurrent down-regulation of STAT3-regulated genes by CD4+ T cells has been eloquently demonstrated—including that of *BCL3*, *PIM1* and *SOCS3* [26]. Such data fuel optimism that cellular biomarkers of STAT3 pathway activation might have clinical value for the development of stratified treatment approaches [27].

Even more tantalizing is the possibility, suggested by our data, that the identified IL-6-mediated transcriptional programme might itself mark a molecular mechanism by which susceptible CD4+ T cells switch to adopt a pathogenic phenotype. Such speculation stems from a functional consideration of the three component signature genes we have identified. BCL3 is an atypical IκB family member which has, until recently, been little studied in human T cell biology [28, 29]. Particularly implicated in the development of T follicular helper cells [30, 31], it appears to represent a common element upon which a range of dysregulated cellular pathways converge [32], and may play a role in restraining the plasticity of the CD4+ T cell effector phenotype [33]. PIM1, one of a family of three serine/threonine-dependent kinases, has been implicated as an early mediator of Th1 commitment [34]; it was recently suggested as a novel therapeutic target in skin psoriasis [35]. SOCS3 is a negative regulator of STAT3 signalling, and whether its up-regulation in early RA reflects a direct failure of this regulatory system is unknown, but it is notable that spontaneous inflammatory arthritis develops in mice following mutation of the molecule’s IL-6 β-receptor binding site [36].

Taken together, it is of interest that the robust three-gene CD4+ T cell signature we now validate parallels the STAT3-dependent transcriptional pattern observed in the malignant Sézary cells of individuals with the leukaemic variant of cutaneous T cell lymphoma, in which up-regulated *PIM1* and *SOCS3* is specifically described [37, 38]. By contrast, all three signature genes are down-regulated in circulating CD4+ T cells of patients with latent tuberculosis infection when compared with those with active infection [39]. The extent to which their induction by IL-6 promotes autoimmunity by sustaining a pathogenic, pro-proliferative cell phenotype—indeed, whether their modulation might favour tolerance induction—warrants concerted investigation. Such studies may reveal targetable disease mechanisms relevant to autoimmune diseases beyond RA alone.

## Supplementary Material

kez003_Supplementary_Data
