# Supplementary material for: Expression of STAT3-regulated genes in circulating CD4+ T cells discriminates rheumatoid arthritis independently of clinical parameters in early arthritis
Source: Rheumatology (Oxford). 2019 Feb 8;58(7):1250–8. doi: 10.1093/rheumatology/kez003 (PMC6587924; doi:10.1093/rheumatology/kez003)
Supplement: kez003_Supplementary_Data [file kez003_supplementary_data.zip › kez003-Suppl_data/Supplementary_Data.docx]

**SUPPLEMENTARY MATERIAL**

**Supplementary Table S1. Baseline clinical characteristics of patients included in meta-analysis according to diagnosis**

|  | **Diagnosis**^1^ | | **p-value**^2^ |
| --- | --- | --- | --- |
|  | **RA**  (n=101) | **Non-RA**  (n=178) |  |
| **Age**, years | **59** (21 - 87) | **51** (17 - 92) | <0.001 |
| **% Female** | **70** | **66** | ns |
| **Symptom dur^n^.** | **12** (2 - 52) | **14** (2 - >52) | ns |
| **TJC/28** | **5** (1 – 20) | **3** (0 – 25) | <0.001 |
| **SJC/28** | **2** (1 – 17) | **0** (0 – 9) | <0.001 |
| **CRP**, g/l | **11** (<5 – 66) | **5** (<5 – 189) | <0.001 |
| **ESR** | **32** (4 – 86) | **9** (1 – 113) | <0.001 |
| **%RF+** | **70** | **8** | <0.001 |
| **%ACPA+** | **67** | **1** | <0.001 |
| **DAS28** | **4.92** (1.99-8.72) | n/a | n/a |
| **Non-RA diagnoses;**  Number (% of 178) |  | PsA 41 (23%)  Other SpA 25 (14%)  Crystal 12 ( 7%)  Other IA 20 ( 11%)  OA 41 (23%)  Other non-IA 39 (22%) |  |

*Values are median (range) unless otherwise stated. ^1^Baseline diagnosis was validated at median 20 months (range 13-25). ^2^Mann Whitney-U test or Chi squared test with Yates’ continuity correction for continuous and dichotomous data respectively. TJC: tender joint count; SJC: swollen joint count; DAS: disease activity score (ESR, 28 joints); PsA: psoriatic arthritis; SpA: spondyloarthropathy; OA: osteoarthritis; IA: inflammatory arthritis.*

**Supplementary Table S2. Normalised expression values of indicated transcripts in early arthritis patient diagnostic groups in meta-analysis.**

| Gene | | | Normalised Expression^1^ | | Fold-change^2^ | P value^3^ |
| --- | --- | --- | --- | --- | --- | --- |
| Symbol | RefSeq | Illumina Probe ID | RA  (n=101) | Non-RA  (n=178) |  |  |
| **BCL3** | **NM_005178** | **ILMN_1710514** | **875** | **579** | **1.51** | **<0.001** |
| **PIM1** | **NM_002648** | **ILMN_1815023** | **4343** | **3123** | **1.39** | **<0.001** |
| **SOCS3** | **NM_003955** | **ILMN_1781001** | **691** | **498** | **1.39** | **0.001** |
| LDHA | NM_005566 | ILMN_1807106 | 7759 | 6762 | 1.14 | <0.001 |
| GPRIN3 | CR743148^4^ | ILMN_1901616 | 457 | 381 | 1.19 | <0.001 |
| MUC1 | NM_001044391 | ILMN_1756992 | 312 | 270 | 1.15 | <0.001 |
| PDCD1 | NM_005018 | ILMN_1806725 | 187 | 161 | 1.16 | <0.001 |
| SBNO2 | NM_014963 | ILMN_1808811 | 248 | 213 | 1.16 | <0.001 |
| IGFL2 | NM_001002915 | ILMN_1790227 | 133 | 125 | 1.06 | <0.001 |
| LOC731186 | XM_001128760 | ILMN_1900154 | 153 | 145 | 1.05 | <0.001 |
| CMAHP | NR_002174 | ILMN_1704084 | 267 | 256 | 1.04 | 0.001 |
| NOG | NM_005450 | ILMN_1652287 | 146 | 160 | -1.09 | <0.001 |

*RefSeq accession numbers and Illumina probe Ids are given (see Supplementary Table 1 for probe sequences). ^1^Median normalised gene expression values presented; ^2^Linearised fold-change relative to non-RA group given; ^3^Mann Whitney-U test. ^4^* *Transcript CR743148 has been retired from NCBI, but the expressed sequence tag corresponds to splice variant(s) within the GPRIN3 gene (chromosome 4.90). Genes demonstrating >1.2-fold differences indicated in boldface.*

**Supplementary Table S3. Confirmation of association between CD4+ T cell gene expression and systemic inflammation**

| Spearman’s rank correlation statistics | | |
| --- | --- | --- |
| Variables | Correlation coefficient | p-value |
| CRP (g/l) vs normalised BCL3 expression | 0.425 | <0.001 |
| CRP (g/l) vs normalised PIM1 expression | 0.464 | <0.001 |
| CRP (g/l) vs normalised SOCS3 expression | 0.548 | <0.001 |

Supplementary figure legend

**Supplementary Table S1. Absence of correlation between PDCD1 or IGFL2 expression with pSTAT3 in paired circulating CD4+ lymphocytes***. Spearman’s Rho correlation coefficients and associated p-values are depicted. Stratification of the cohort according to diagnostic category had no impact on the outcome of this analysis.*
