# Supplementary figures and images for: Expression of STAT3-regulated genes in circulating CD4+ T cells discriminates rheumatoid arthritis independently of clinical parameters in early arthritis
Source: Rheumatology (Oxford). 2019 Feb 8;58(7):1250–8. doi: 10.1093/rheumatology/kez003 (PMC6587924; doi:10.1093/rheumatology/kez003)

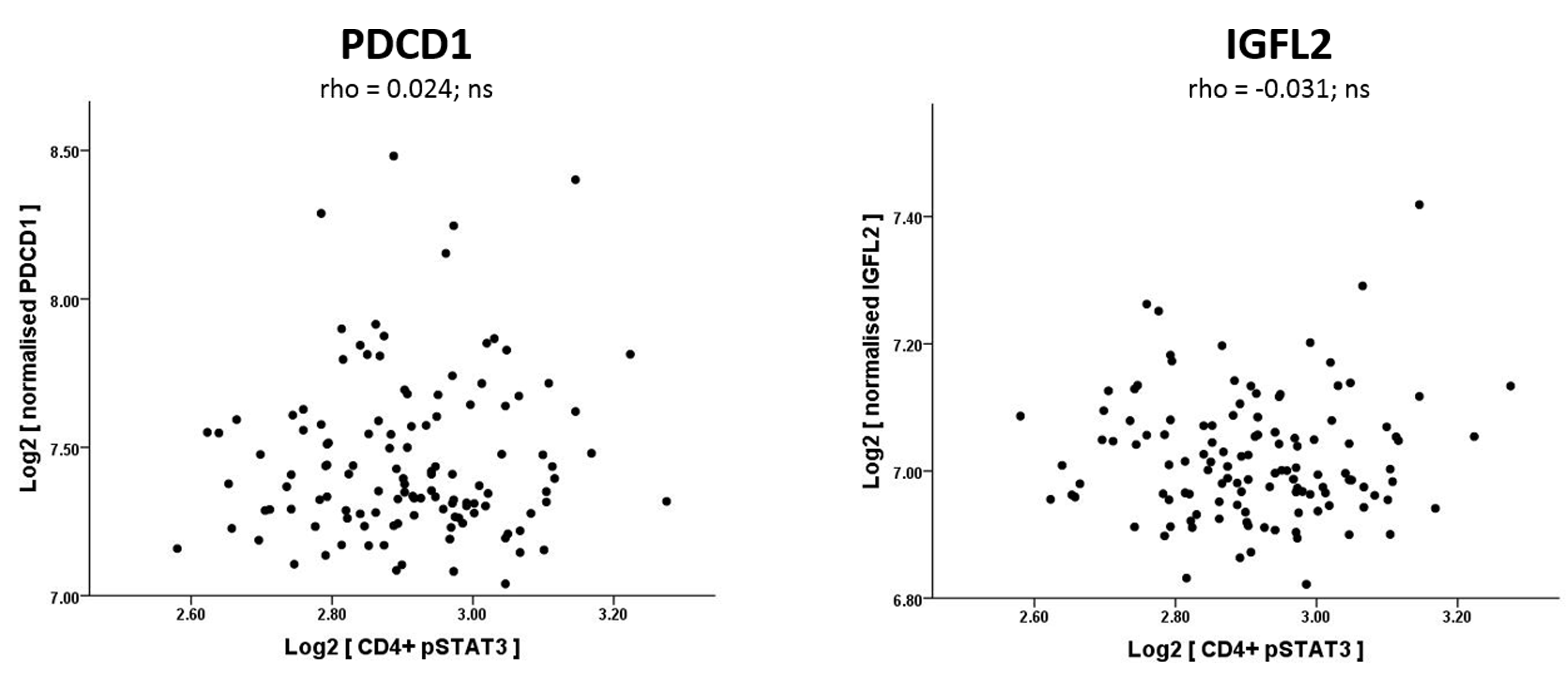

Supplement: kez003_Supplementary_Data [file kez003_supplementary_data.zip › kez003-Suppl_data/Supplementary_Figure.tif]
